# A catalogue of 863 Rett-syndrome-causing *MECP2* mutations and lessons learned from data integration

**DOI:** 10.1038/s41597-020-00794-7

**Published:** 2021-01-15

**Authors:** Friederike Ehrhart, Annika Jacobsen, Maria Rigau, Mattia Bosio, Rajaram Kaliyaperumal, Jeroen F. J. Laros, Egon L. Willighagen, Alfonso Valencia, Marco Roos, Salvador Capella-Gutierrez, Leopold M. G. Curfs, Chris T. Evelo

**Affiliations:** 1 Department of Bioinformatics - BiGCaT, NUTRIM School of Nutrition and Translational Research in Metabolism, MHeNS School of Mental Health and Neuroscience, Maastricht University, Maastricht, The Netherlands; 2 GKC - Rett Expertise Centre, Maastricht University Medical Center, Maastricht, The Netherlands; 3 Department of Human Genetics, Leiden University Medical Center, Leiden, The Netherlands; 4 Barcelona Supercomputing Centre (BSC), Barcelona, Spain; 5 Catalan Institution for Research and Advanced Studies (ICREA), Barcelona, Spain

**Keywords:** Genetics research, Neurological disorders, Data integration, Disease genetics

## Abstract

Rett syndrome (RTT) is a rare neurological disorder mostly caused by a genetic variation in *MECP2*. Making new *MECP2* variants and the related phenotypes available provides data for better understanding of disease mechanisms and faster identification of variants for diagnosis. This is, however, currently hampered by the lack of interoperability between genotype-phenotype databases. Here, we demonstrate on the example of *MECP2* in RTT that by making the genotype-phenotype data more Findable, Accessible, Interoperable, and Reusable (FAIR), we can facilitate prioritization and analysis of variants. In total, 10,968 *MECP2* variants were successfully integrated. Among these variants 863 unique confirmed RTT causing and 209 unique confirmed benign variants were found. This dataset was used for comparison of pathogenicity predicting tools, protein consequences, and identification of ambiguous variants. Prediction tools generally recognised the RTT causing and benign variants, however, there was a broad range of overlap Nineteen variants were identified that were annotated as both disease-causing and benign, suggesting that there are additional factors in these cases contributing to disease development.

## Abstract (summary)

**Table:** 

Measurement(s)	Rett syndrome • phenotype • MECP2 Gene
Technology Type(s)	digital curation • network analysis
Sample Characteristic - Organism	Homo sapiens

Machine-accessible metadata file describing the reported data: 10.6084/m9.figshare.13359476

## Background and Summary

Rett syndrome (RTT) is a rare neurological disorder first described in 1956 by Andreas Rett occurring predominantly in females^1^. In most cases, the disorder is caused by a loss-of-function variation on the X-bound gene for MECP2 (methyl-CpG-binding protein 2)^2,3^. Phenotypic severity is thought to vary due to X-inactivation, mosaicism, severity of the variation (loss of function vs. impaired function), genetic background (ref. ^4^ and literature cited therein) and environmental factors.

On the molecular level, the MECP2 protein recognizes and binds to specific methylated and hydroxymethylated DNA regions, and attracts several other proteins to form a transcription repression block. This block makes the DNA sequence accessible for histone deacetylases, which increases the packing density of these regions, reducing their transcriptional activity^5^. Several metastudies on omics data revealed that the influence of MECP2 affects dominantly dendritic connectivity, synapse function, glial cell differentiation, mitochondrial function, mRNA processing and translation, inflammation, and cytoskeleton^6–8^.

The MECP2 protein has five different domains: N-terminal domain (NTD), methyl-DNA binding domain (MDB), transcription repressor binding domain (TRD), intermediate domain between methyl-DNA binding and transcription repressor binding domain also called interdomain (ID), C-terminal domain (CTD)^9^. Ballestar and coworkers found that *MECP2* variations that slightly decrease the specific recognition of the binding site on DNA are able to cause RTT^10^. The majority of RTT causing missense variations are found in the methyl-DNA binding domain, but RTT causing variations have been found in all parts of the protein^11^. Some studies have found a distinctive correlation of phenotype severity and variation type^12^, while others found a rather small or insignificant correlation^13–16^.

Due to the rareness of RTT (prevalence about 1:10.000, ref. ^17^), it is important to share and communicate information about disease causing variations to increase the success of identifying genetic causes. In a previous study, we investigated the status of RTT genotype-phenotype databases and the methods that different resources use to share newly identified genetic variants on the example of RTT^18^. Thirteen different genotype-phenotype databases were identified that are used to collect and share genetic variants annotated with observed or predicted effects. Our main conclusion was that databases store and provide information in very different ways, such that now it is technically infeasible to query multiple databases and combine the results in an efficient and automated way. In line with the IRDiRC aims for rare diseases (http://www.irdirc.org/about-us/vision-goals/), the bioinformatics infrastructure should contribute to store, curate and make data about known disease causing and benign variations available. Therefore, the interoperability of these databases needs to improve to be able to efficiently use their contents in combination.

In this study, we show how to integrate the available RTT genetic and phenotypic data across multiple databases and use the integrated data for further analysis about RTT, in order to investigate variant abundance and distribution and to test variant effect prediction algorithms. We followed the FAIRification workflow^19^ to make the data more findable, accessible, interoperable, and reusable for computer processing. In line with the FAIR data point specification, a combination of DCAT and Re3Data vocabularies were used to describe the data set [https://github.com/FAIRDataTeam/FAIRDataPoint-Spec/blob/v0.1.0/spec.md]. The resulting ‘FAIR data point’ refers to two distribution formats: one in RDF and one in CSV. RDF was used to create a self-describing, machine interpretable version of the data using existing global ontologies. The resulting datasets (CSV) are also shared on Figshare (see DOI in results). To our knowledge, the dataset created and used in this study is the largest collection of annotated disease-causing and benign *MECP2* variants available at this moment, and may help researchers investigate and test disease models.

## Methods

### Workflow of genetic variant data integration

#### Data selection and retrieval

**Fig. 1:**
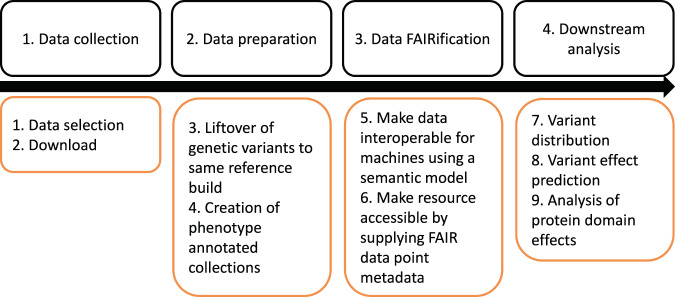
Schematic drawing of the workflow of this study: data collection, preparation, FAIRification and downstream analysis.

In a recent study^18^, we identified thirteen genotype-phenotype databases containing RTT-specific *MECP2* variation data. We evaluated each of these for specific requirements for data integration. Data should be 1) available and permitted to be re-used and redistributed, 2) the given description of genetic variants should be for an unambiguous variation. The latter means that the exact position (chromosome build and location) as well as the variation of the genetic variants are available or retrievable by conversion, thus, they can be described using the HGVS nomenclature. For this study, we selected eight databases and downloaded all *MECP2* genetic variants with available linked phenotype information from each of these databases: ClinVar^20^, https://www.ncbi.nlm.nih.gov/clinvar/, DECIPHER^21^, https://decipher.sanger.ac.uk/, EVA (http://www.ebi.ac.uk), EVS (http://evs.gs.washington.edu), ExAC^22^, http://exac.broadinstitute.org/, KMD (https://kmd.nih.go.kr), LOVD^23^, *MECP2* collection: https://databases.lovd.nl/shared/genes/MECP2), and RettBASE^24^, http://mecp2.chw.edu.au/. Additionally, an anonymized dataset from local RTT patients was included (Maastricht Rett dataset, permission granted by Niet-WMO verklaring 2018-0597, Maastricht University METC approval). Either the integrated download function was used to get the data or data was extracted from HTML (see the availability of download functions in ref. ^18^. Figure 1 shows the data processing (step 1–3) and analysis (step 4) workflow of this study.

#### Liftover to enable compatible genetic variant description formats

The *MECP2* genetic variant descriptions from the different sources were made compatible and therefore comparable by application of the HGVS nomenclature and the same reference sequence. This is the first step to make the data interoperable. For this, we used the reference sequence for chromosome 23 (X) NC_000023.11, which is part of the current human genome reference assembly (GRCh38). Genomic descriptions were used to ensure that variations in and outside the gene region (exonic, intronic, up- and downstream) were included. The process of re-describing all variants with the HGVS nomenclature using the same reference build, liftover, was done by using the Mutalyzer position converter webtool [https://mutalyzer.nl/]^25^. Mutalyzer can perform a conversion between different reference sequences and categories (e.g. complete genomic regions NC and mRNA NM) but requires nomenclature-compliant input. Manual correction was performed on genetic variant descriptions that did not have the complete and correct format for conversion but provided enough information to correct the format.

#### Creation of phenotype annotated collections

Genetic variants were assigned by their linked phenotype information to three different categories: 1) RTT causing (verified by identification as disease causing variant according to the requirements of the databases) (data from DECIPHER, Maastricht Rett dataset, ClinVar, RettBase and KMD), 2) benign (verified by finding them in a healthy control subject) (data from ClinVar and RettBase), and 3) unknown evidence (only pathogenicity prediction scores provided by database) (EVS, EVA, LOVD, ExAC). The exact annotation selection criteria for the RTT causing dataset based on the databases individual annotations are given in Sup. Table 1. These lists are collected and used for further analysis.

#### Data FAIRification

We made the prepared genetic variant and phenotype data more Findable, Accessible, Interoperable, and Reusable for humans and computers following the FAIR guiding principles^26^. The data was made machine-readable (in RDF format) using a semantic data model (see below) and a general-purpose FAIRifier tool^27^ based on the OpenRefine data cleaning and wrangling tool (http://openrefine.org/) and an RDF plugin (https://github.com/stkenny/grefine-rdf-extension). Similarly, machine-readable metadata (information about the data) was generated using the Metadata Editor^27^.

We applied and extended the semantic data model of a genetic variant described in ref. ^28^ to convert the prepared data to RDF. The model is available on GitHub (https://github.com/LUMC-BioSemantics/rett-variant) and describes the important data elements of the datasets: 1) the genetic variant: HGVS nomenclature, start/end position of the variation, and genome build, and 2) the phenotype information that describes whether a variant is thought to be RTT causing, benign or unknown.

### Downstream analysis examples

#### Network analysis of data distribution in RTT databases

To analyse the distribution of *MECP2* variations in the RTT databases, a network was created where the nodes represent databases and the node size the number of available *MECP2* variations. The thickness of the lines connecting the databases indicate how many *MECP2* variations they share. Network visualization and analysis software Cytoscape^29^ was used for this purpose.

#### Variant annotation and characterization by genomic features

To characterize all the collected *MECP2* variants, we developed an automatic analysis pipeline for variant annotation. We used the HGVS corrected variants to integrate custom scripts with HGVS conversion tool from https://github.com/counsyl/hgvs and generated VCF files for annotation within an automated pipeline available at https://gitlab.bsc.es/inb/fair-rett. Afterwards, we proceeded to annotate variants with Ensembl Variant Effect Predictor, VEP^30^ v94 using the GRCh38 assembly, selecting all available features, plus optional plugins to estimate variant pathogenicity (i.e., PolyPhen^31^, SIFT^32^, MetaLR^33^, CADD^34^, FATHMM-MKL^35^ from dbNSFP and dbscSNV scores^36^) both in coding and splicing regions.

The resulting VEP annotated data was processed with R scripts, available at https://gitlab.bsc.es/inb/fair-rett, to compare RTT causing and benign variants as subsets, and to generate summary statistics for these. The scripts allow to compare and visualize the two classes in terms of any of the available VEP annotation features, (e.g. variant frequency in the population, estimated variant consequence, and conservation score of the genomic location). Using this we compared the two datasets of RTT causing and benign variants by pathogenicity scores, impact (i.e. estimation of the consequence of each variant on the protein sequence), variant frequency, and genomic location. Because a few variations appear both as RTT causing and benign, we represented this subset of variants as a third class (“both”) in all visualizations.

Finally, we focused on exonic missense variants and used VEP information about the amino acid change and position within the MECP2-e2 transcript to visualize the variation distribution across protein domains and conserved regions (as described in ref. ^37^). This allowed us to make a finer characterization of differential distribution of RTT causing and benign variants across MECP2 domains.

## Data Records

The machine-readable metadata was made available on a FAIR Data Point^38^ (https://github.com/FAIRDataTeam/FAIRDataPoint-Spec) available via: http://purl.org/biosemantics-lumc/rettbase/fdp. The FAIR Data Point metadata provides URIs that resolve to the RDF and CSV files for each of the nine sources on Figshare (10.6084/m9.figshare.c.4769153.v2)^39^.

## Technical Validation

### Data integration challenges identified

We encountered several challenges while integrating data from the different RTT databases: 1) different descriptions of genetic variants were used, 2) liftover process and limitations in automated liftover, and 3) findability of terms of use/re-use, detailed below.

1) For the descriptions of genetic variants, the most commonly used nomenclature was HGVS. HGVS still comes in different, correct, flavours, e.g. using genomic or cDNA positions or different (versions of) reference sequences, which still need conversions from one to the other_,_ using for instance Mutalyzer. The other most common standard was the RS number (reference SNP identifier, from dbSNP). These are usually linked to loci and can therefore not be used as unambiguous identifiers for a variant. Databases that give only RS identifiers were therefore not included in further analysis. The same problem occurred with the annotation of diagnosis and/or phenotypes. As described before in ref. ^18^, only a few databases link original diagnostic information to the genetic information, and whenever it was provided, this information was presented using different formats or definitions.

2) For the liftover to one common, comparable variant description (GRCh38 (hg19)), genomic position) Mutalyzer was used. It can be used programmatically via API (Application programming interface) or via Graphical User Interface (GUI). After liftover to HGVS nomenclature it was possible for the majority of variants (90.7–100% per dataset) to use Mutalyzer without further curation (Table 1). Nevertheless, for up to 9.3% of the variations in a dataset (Maastricht Rett dataset, the average was 4.3%, Table 1) the data needed curation due to typos, incorrect nomenclature (e.g., symbols which are not in the official nomenclature), or outdated/historic position description (e.g., Genbank variation description nomenclature). Mutalyzer itself cannot deal with insertions of a number on unknown base pairs (e.g., ins3 instead of insATT), round brackets () to indicate uncertainty (they are gone after translation while square brackets [] to indicate different alleles or group alleles are fine), asterisk * to indicate stop (protein) according to the official HGVS nomenclature. These variations required manual curation, e.g. changing round brackets to square brackets, use Mutalyzer to do the liftover, changing square brackets back to round brackets. Furthermore, it is currently not possible to do a direct liftover from one genomic reference sequence to another (e.g., NC_000023.10:g.153282026 G > A to NC_000023.11:g.154016575 G > A) due to the size of the reference sequence. At the moment, this must be done in two steps via transcript (NC - > NM - > NC).

**Table 1:** Overview for the different databases, their phenotype annotation format, number of available *MECP2* variants, and data liftover success rates using automated (Mutalyzer) and manual curation.

Phenotype annotation format	Database	Number of *MECP2* or RTT variations	Number of variations meeting annotation criteria	Number of variations with sufficient genetic annotation			
				Using Mutalyzer		After manual curation	
				#	%	#	%
Phenotype	DECIPHER	34	25	25	100	25	100
Diagnosis	Maastricht Rett dataset	429	428	388	90.7	393	91.8
	ClinVar	1,134	743	681	91.7	726	97.7
	RettBase	4,705	3,986	3,798	95.3	3,980	99.8
	KMD	35	35	35	100	35	100
Pathogencity scores	EVS	190	190	190	100	190	100
	LOVD	808	808	738	91.3	804	99.5
	EVA	4,226	4,226	4,193	99.2	4216	99.8
	ExAC	599	599	559	93.3	599	100
TOTAL		12,158	11,040	10,607	average 95.7%	10,968	average 98.7%

3) The permission to reuse and redistribute was difficult to find for some databases (RettBase, KMD).

### Size and content of the FAIR dataset

#### Number of disease causing and benign *MECP2* genetic variants available

Based on the thirteen genotype-phenotype databases identified in ref. ^18^, the inclusion criteria for this study were not met by DisGeNET, dbSNP, dbVAR, Café Variome, and HGMD. DisGeNET, dbSNP and dbVAR did not provide unambiguous descriptions of variations as the RS identifier only indicates a location of polymorphism and needs evaluation of the, sometimes ambiguous, additional information about the nucleotide change. Café Variome provided only protein change information, which, although very relevant itself, cannot be translated back to an unambiguous genetic change. HGMD, the only commercial database, did not allow re-use and re-distribution of the content. The eight databases that did fulfil our inclusion criteria and data previously anonymized from local RTT patients were used in this study (see Table 2). At the time of research, in total 12,158 *MECP2* variation entries were found in these databases. The databases contained between 34 (DECIPHER) and 4,706 (RettBASE) *MECP2* variations (Table 2). Between 15% and 100% of these variations were unique database entries (occur only once in one single database). Multiple entries of one variation were found frequently in disease specific databases, giving an indication of the abundance of this variant and confirming its pathogenicity. In total we identified 4,573 RTT causing *MECP2* variants (of which 863 were unique) that annotate genetic information with diagnosis (RettBase, ClinVar, Maastricht Rett dataset, KMD) and/or clear phenotype descriptions (DECIPHER) clearly stating that they cause RTT (or similar e.g., X-linked mental retardation) (intake criteria Sup. Table 1). We identified 617 benign *MECP2* variants, of which 209 were unique, from two of the databases that annotate with diagnosis information (RettBase and ClinVar). These were clearly stated to be benign. Nineteen variants were found annotated both as RTT causing and benign (Sup. Table 2).

**Table 2:** Numbers of total and unique *MECP2* variations in each database.

Database	Number of total *MECP2* variation entries	Number of unique *MECP2* variations		Number of unique variations which occur only in this database
		#	% of total *MECP2* variation entries	
EVA	4,226	4,192	99.2	3,329
LOVD	808	802	99.3	144
RettBase	4,705	740	15.7	209
ExAC	599	599	100.0	40
ClinVar	1,134	716	63.1	126
EVS	190	95	50.0	1
Maastricht Rett dataset	429	68	15.9	34
KMD	35	35	100.0	9
DECIPHER	34	23	67.6	2

In total, we collected 12,158 *MECP2* variants, which resulted in a collection of 10,968 (5,038 unique) curated and integrated variants. Out of the 10,968 curated *MECP2* variations only eleven occur in more than 1% of all database entries, and these account for 53.7% of all database entries (data not shown).

The 863 unique RTT causing variations are distributed over 4,573 database entries. Also here, only twelve variations are found in more than 1% of all database entries (Table 3) and these twelve make in total 60% of the database entries. The most abundantly found *MECP2* variations were found in seven of nine databases (Table 3). The majority (eight) of these are C > T transitions at CpG hotspots^40^. These eight *MECP2* hotspot variations contribute to 49.7% of all *MECP2* variation entries. The most abundant *MECP2* variation in this dataset is NC_000023.11:g.g.154031355 G > A (NM_004992.3:c.473 C > T, NP_004983.1:p.(Thr158Met)) with 463 counts (Table 3). In total 54% of RTT causing variations are a deletion, 9% insertion, 37% substitution, and 9% duplication. Many of the database entries contain multiple variations (e.g., a deletion and insertion) on the same or different chromosomes. 452 RTT causing variations have only one single database entry and of these 269 are a deletion, 43 insertion, and/or 153 substitution.

**Table 3:** Most abundant RTT causing variants in this study.

Genomic position^†^	count	%	cDNA^‡^ and protein change §	Effect and previous reports
g.154031355 G > A	463	10.1	c.473 C > T, p.(Thr158Met)^¶^	Missense variation^24,43,44^
g.154031326 G > A	409	8.9	c.502 C > T, p.(Arg168*)^¶^	Nonsense variation, leading to truncation^24,43,44^
g.154031065 G > A	345	7.5	c.763 C > T, p.(Arg255*)^¶^	Nonsense variation, leading to truncation^24,43,44^
g.154031020 G > A	309	6.8	c.808 C > T, p.(Arg270*)^¶^	Nonsense variation, leading to truncation^24,43,44^
g.154030948 G > A	281	6.1	c.880 C > T, p.(Arg294*)^¶^	Nonsense variation, leading to truncation^24,44^
g.154030912 G > A	279	6.1	c.916 C > T, p.(Arg306Cys)^¶^	Missense variation^24,43,44^
g.154031431 G > A	249	5.4	c.397 C > T, p.(Arg133Cys)^¶^	Missense variation^24,44,53^
g.154032268 G > A	161	3.5	c.316 C > T, p.(Arg106Trp)^¶^	Missense variation^24,44^
g.154031373 G > C	80	1.7	c.455 C > G, p.(Pro152Arg)	Missense variation^54^
g.154031022delC	67	1.5	c.806delG, p.(Gly269fs)	Frameshift deletion leading to missense^41^
g.154030621_154030664del44	50	1.1	c.1164_1207del44, p.(Pro389*)	Frameshift deletion leading to truncation
g.154030631_154030671del41	49	1.1	c.1157_1197del41, p.(Leu386fs)	Deletion leading to frameshift

RefSeq ^†^NC_000023.11, ^‡^NM_004992.3, ^§^NP_004983.1.

^¶^one of the eight hotspot variations^40^.

### Distribution of the variants across databases

Table 2 shows the number of unique *MECP2* variations for each investigated database. The different databases contain very different numbers of unique *MECP2* variations. The number of unique MECP2 variations in a database gives an indication whether it is a database focusing on collecting pathogenic variations (RettBase, ClinVar, Maastricht Rett dataset, DECIPHER) (exception KMD) or general population sequencing results (no disease annotation) (EVA, LOVD, ExAC) (exception EVS). LOVD, for example, lists all different variations and provides background information about the abundance of one variation in the variations’ information sheet. RettBase also gives the reference where a specific entry is from. From Table 2 it also becomes clear that every database has unique *MECP2* variations, which are found in no other database. The number of such unique variants differ between 3,329 (EVA) and one (EVS).

**Fig. 2:**
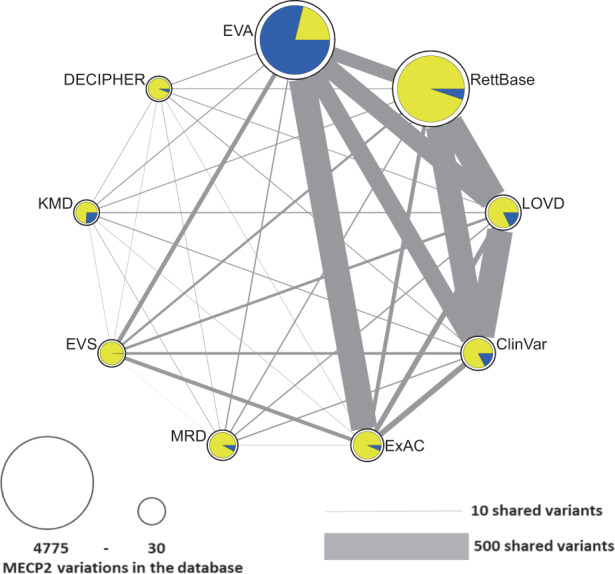
Network illustrating the number of unique and overlapping *MECP2* variations within and between nine Rett syndrome databases: DECIPHER, Maastricht Rett dataset (MRD), ClinVar, Rettbase, KMD, EVS, LOVD, EVA, and ExAC. Each node (circle) represents a database. The node size correlates with the number of variants (between 30 and 4775), the edge thickness correlates with the number of overlapping/shared variants between the two databases (between 0 and 500). The colour of the charts in the nodes represent the proportion of unique variants (blue) versus variants shared with other databases (yellow).

Figure 2 shows the size of *MECP2* variation collections in the different databases, their shared and their unique variations. There are databases that focus on collections of genome and/or exome sequencing data of mostly healthy individuals (EVA, EVS, ExAC), curated collections of disease causing variants (LOVD, RettBase, ClinVar, Decipher), and hospital derived collections (KMD, Maastricht Rett dataset). The overlap or shared *MECP2* variations between databases can be explained by the occurrence of this variation in multiple patients, data exchange between databases, or by recruitment from the same resources. For instance, ExAC and LOVD share 559 unique variants, LOVD and ClinVar 546, LOVD and RettBase 512, RettBase and ClinVar 504.

### Advantages of making the *MECP2* genetic variant data FAIR

The FAIR guiding principles have emerged from analysing the general, and often repeated, process that data scientists go through when preparing data from multiple sources for data integration and analysis. The *MECP2* genotype-phenotype data from this study were retrieved from nine heterogeneous resources, which we prepared for analysis by making them more FAIR. This was first and foremost done to enable integration of the data for analysis as correctly as possible, which also facilitates integration with other interoperable data such as protein functionality data from, for instance, UniProt, NextProt or Phyre databases. Another reason was to ensure reusability of the integrated data for other research studies. Note, all the FAIRified resources allow redistribution.

The FAIRified data was described with machine-readable metadata and distributed at a new location, which prospectively allows other researchers to reuse this data. Thus, as data users, we made the data FAIR after retrieving them from their respective distributions. This was necessary, because the way that the data were provided by the different sources was not sufficiently uniform for machines to integrate multiple sources. The disadvantage of leaving the implementation of FAIR principles to data consumers is that they are more likely to make mistakes in the interpretation of the meaning of the data, which may not be the same as the sources. Ideally, data are made FAIR at the source to minimize that risk and optimize transparency. This would have allowed us to directly use the data in automated workflows that can be run regularly to update our findings.

## Usage Notes

### General use of the data

#### The CSV formatted files

The CSV formatted files contain in the first column genetic variants generated and used in this effort, which were described with the HGVS nomenclature, a common format to describe genetic variants. The second column indicates the source (name of the database) and the third column classifies the variations into RTT causing, benign or NA (not applicable). Any analysis or visualization tool that can deal with HGVS formatted files like VCF can work with this format.

#### The RDF files

The RDF output of this dataset is serialized in the Turtle format. To use these RDF files, we need to upload it to a triple store, which is a special database designed to store and query RDF files. We can use the SPARQL query language to query the RDF files. We linked our RDF to external RDF data sources such as Ensembl RDF and Orphanet Rare Disease ontology. Within a SPARQL query we can also exploit these links to do further integration queries.

#### Visualization in genome browsers

Broadly used genome browsers, e.g. UCSC accepts HGVS variant descriptions in their interactive interface for visualization purposes, e.g., see the result [Link] of NC_000023.11:g.1000000 C > T. In addition, conversion from the CSV formatted file to a “BED detail” formatted file [Link] should be relatively straightforward prior to its ingestion by the genome browsers. This alternative format can also be used in most modern genome browsers.

### Biological questions answered using this data

#### Variant pathogenicity prediction vs. curated datasets

**Fig. 3:**
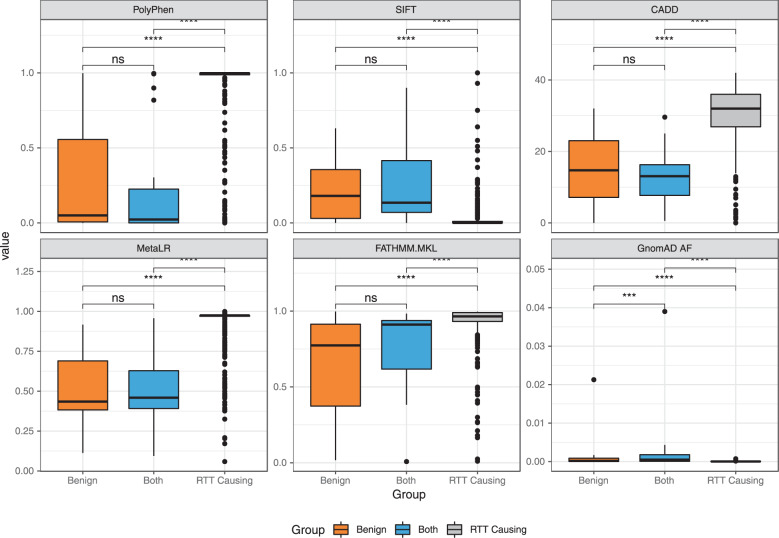
Boxplots comparing prediction score value distribution calculated by different tools from the benign, both and RTT causing *MECP2* genetic variants. The effect prediction was done based on conservation score (PolyPhen), four pathogenicity scores (SIFT, CADD, MetaLR, and FATHMM.MKL), and the variant allele frequency in the GnomAD dataset.

To explore differences between RTT causing and benign *MECP2* genetic variants we analyzed the annotated results from VEP (see Methods) from six descriptive features (Fig. 3). We chose to visualize the obtained scores about conservation (i.e., PolyPhen), pathogenicity estimation scores (i.e., SIFT, CADD, MetaLR, FATHMM-MKL), and the variant frequency in normal population from GnomAD^22^ (i.e., GnomAD_AF).

We classified variants by benign, RTT causing and “both”, as we identified a subset of 19 variants appearing in both datasets. The detailed list of the *MECP2* variations, which occur in both, can be found in Sup. Table 2. Overall, we see expected results: the RTT causing variants were found to be in positions significantly more conserved than the benign or both variants (Fig. 3, PolyPhen (Wilcoxon test)), as well as less frequent than benign variations even though, all variants presented here are not abundant in the normal population (Fig. 3, GnomAD_AF). Analysis of the obtained estimation of pathogenicity from multiple scores (Fig. 3 panels SIFT, CADD, MetaLR and FATHMM-MKL), shows that RTT causing variants are on average predicted as more damaging than the benign and both variants (p < 0.0001 in all cases after applying Wilcoxon test). Note that SIFT associates more pathogenic variants to lower scores, whereas CADD, MetaLR and FATHMM-MKL associates more pathogenic variants to higher scores. MetaLR is better than the other three pathogenicity scores in distinguishing benign and RTT causing variant types. This may be because this novel meta-score integrates more features than the other three prediction tools, amongst other pathogenicity scores and frequency information.

The pathogenicity estimates of the both group place the variants between the benign and RTT-causing in three out of five predictions, while in the other two give a prediction more similar to the benign group.

#### Distribution of pathogenic and benign missense variations to protein domains

**Fig. 4:**
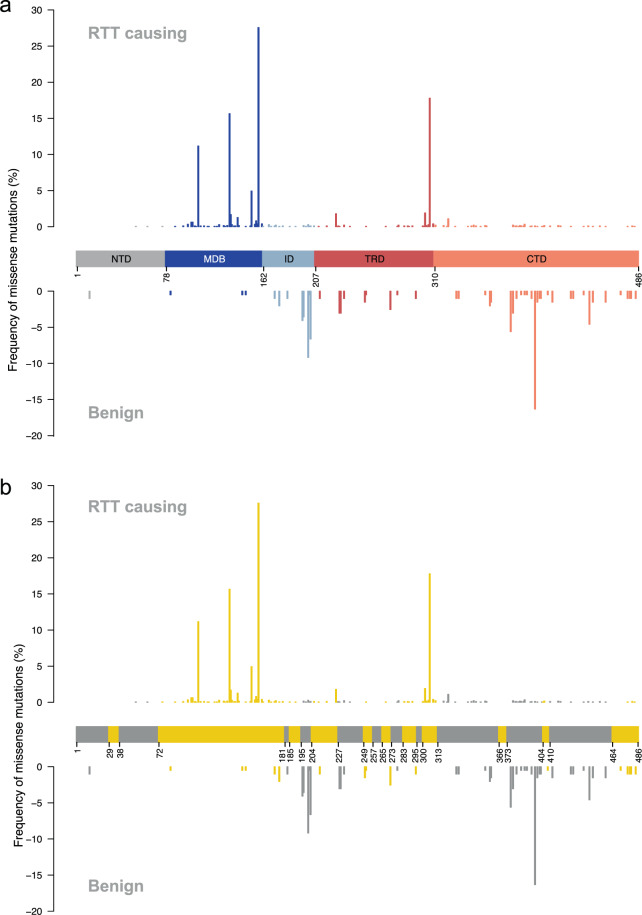
Distribution of RTT causing and benign *MECP2* missense variations. Amino acid positions correspond to isoform MECP2-e2 (the result of translation initiated at exon 2). Frequency is represented as the percentage of missense variations falling in each position, from the total of missense variations in cases or controls. In a) each MECP2 domain is coloured differently, while in b) conserved deletions are coloured in yellow. Domain abbreviations: N-terminal domain (NTD), methyl-DNA binding domain (MDB), interdomain (ID), transcription repressor binding domain (TRD), C-terminal domain (CTD).

In this experiment the position of RTT causing and benign missense variants in different domains and conserved regions of *MECP2* are compared (Table 4 and Fig. 4). Most RTT causing missense variations are found in the methyl-DNA binding domain (MDB) (68.3%) and in the transcription repressor binding domain (TRD). However, at lower frequencies, RTT causing missense variations can also be found in the other domains. The benign variants are most frequent in the C-terminal domain (55.1%) and the interdomain (28.1%), but can likewise also be found in the other domains at lower frequencies. The distribution across the conserved regions of *MECP2* shows that 93.6% of the missense RTT causing variants are found in conserved regions while only 16.3% of the benign variants are found in conserved regions.

**Table 4:** Location of RTT causing and benign missense variants in different domains and conserved regions of MECP2.

		Domain length (% of total)	RTT causing	Benign
			% of missense variations per region	
Domains	N-terminal domain	78 (16.0)	0.2	1
	Methyl-DNA binding domain	84 (17.3)	68.3	1.5
	Interdomain	45 (9.3)	1.9	28.1
	Transcription repressor domain	103 (21.2)	24.1	14.3
	C-terminal domain	176 (36.2)	5.5	55.1
Conserved regions			93.6	16.3

Regarding mutations affecting the protein sequence, in 1350 cases, which is about half of the RTT-causing single nucleotide mutations, the variations are truncating, changing an Arginine into a stop codon. Also frequently, Arginine is changed into a Cysteine (533) or Tryptophan (179) which are major changes in protein 3D structure. The average BLOSUM62 value of all amino acid changes for the RTT causing dataset is −1.8. For the benign *MECP2* variations, the most abundant variations are silent ( = not amino acid changing), coding for Serine (65), Threonine (44) and Proline (40). The most abundant amino acid change is Glutamic acid to Lysine (33) and the average BLOSUM62 value of all amino acid changes indicates with −0.3 less severe consequences for the protein structure than the RTT causing group.

#### Added-value of integration of data across different sources

This is to our knowledge the first study that integrates genetic variation data from multiple databases on *MECP2*. Despite best efforts of individual sources to reach the largest possible coverage, our results demonstrate that the number of usefully annotated variants increases when databases are combined. The greatest advantage of the integrated approach is therefore that more variants become available for further research and diagnosis. This is especially interesting for rare diseases which have relatively small study populations. By mapping to a common reference sequence, the information of different sources becomes comparable and we are getting closer to the “true” number of variants known. In this study, we were able to increase the previously estimated numbers of a few hundred RTT causing unique sequence variations to 863. However, databases, at least the active ones, get regular updates and input of data. In the time from the beginning of this study the number of variants in e.g. RettBase increased from 4,738 (March 2018^18^) to 4,757 (November 2018) to 4,806 (NM_004992.3, April 2020). Consequently, the number of 863 known RTT causing variants is likely outdated when this study is published. We argue that it is unrealistic to assume that any single database will ever be completely comprehensive, unless it automatically pulls in updates from other databases. A possible contribution to the solution of this problem would be to create the combined list of pathogenic variants by automated workflows that find and summarize data from across databases on demand or continuously. To make that possible we need to standardize how databases provide data for machine processing. The role of FAIR data principles to achieve this is discussed later in more detail.

This integrated dataset gives the possibility to study abundance and prevalence of certain variations in a larger population than any of the study populations published before. There are several studies on relatively small^41,42^ or large populations (e.g^43,44^.) that have published their data in the previous years. Study ref. ^43^ analysed 301 different *MECP2* alleles in a French population and found 69 different variations, which cause 64% of RTT. They identified NP_004983.1:p.R168*, R255*, R270*, T158M, and R306C (Table 5) as the most abundant variations and 59 variations were found in only one or two patients. In the list from the US national history study (819 participants^44^) the variations R106W, R133C, T158M, R168*, R255*, R270*, R294*, and R306C were responsible for more than 60% of RTT. The *MECP2* variation content of RettBase was analyzed recently by ref. ^24^ and the following eight hotspot variations are responsible for a total of 47% of RTT cases (of total number of *MECP2* entries was at that time 4668, disease causing and benign): R106W, R133C, T158M, R168*, R255*, R270*, R294*, and R306C. ref. ^3^ provides information about eleven more datasets from different countries.

**Table 5:** Comparison of most abundant *MECP2* variations in different studies.

Sample size and citation	Variations mentioned in studies (abundance in % if known)							
4573 variations annotated with RTT causing (this study)	Thr158Met (10.1)	Arg168* (8.9)	Arg255* (7.5)	Arg270* (6.8)	Arg294* (6.1)	Arg306Cys (6.1)	Arg133Cys (5.4)	Arg106Trp (3.5)
301 RTT patients^43^	Thr158Met (7.8)	Arg168* (11.5)	Arg255* (10.9)	Arg270* (10.5)		Arg306Cys (6.8)		
819 RTT patients^44^	Thr158Met (11.0)	Arg168* (10.4)	Arg255* (11.0)	Arg270* (5.5)	Arg294* (6.3)	Arg306Cys (6.9)	Arg133Cys (3.8)	Arg106Trp (3.1)
RettBase 4668 total entries^24^	Thr158Met	Arg168*	Arg255*	Arg270*	Arg294*	Arg306Cys	Arg133Cys	Arg106Trp

RefSeq: NP_004983.1:p.

Although our study resulted in a different ranking of the eight hotspots, we could confirm these as the most abundant ones which occur in our dataset in 54.6% of all RTT causing database entries. All eight hotspot mutations are C > T transitions leading in seven of eight cases to a change from Arginine to a stop codon, Cysteine or Tryptophan which are changes with a high probability to change the 3D structure of the protein. The special vulnerability of certain Cytosine positions to errors in base excision repair was described before^45^.

In our integrated dataset most pathogenic mutations in *MECP2* occur in the methyl-DNA or transcription repressor binding domain. This finding has been described and confirmed before^24,46–48^. The functionality of the methyl-DNA binding domain is reported to be extremely sensitive to changes^46^. The importance of the domain also shows from the observation that a construct consisting only of methyl-DNA binding and transcription repressor domain could preserve some basic functions of MECP2^49^. There is also a clear distinction between conserved and non-conserved regions. As expected, disease-causing mutations occur much more often in the conserved regions. However, the data shows clearly that mutations in all domains, both conserved and non-conserved regions, can cause RTT. The open question here remains how much influence does a particular mutation have and how much is contributed by other genetic aspects or environmental influences. This question becomes more important considering the discovery of variants that in one individual can be benign and RTT causing in another.

#### How can the same variation be benign AND cause RTT in different individuals

The majority of the *MECP2* genetic variations, which are described as RTT causing in one, and benign in another database entry, are predicted to be benign (Fig. 3). Possible explanations why a variant can be disease causing in one individual and benign in another could be due to the location of the gene on the X chromosome which may result in a subclinical phenotype in females but a fully-fledged RTT in male patients. The sex of patients is usually not given in these genotype-phenotype databases. In addition, X-inactivation patterns^50^ and genetic background related to other participating genes in *MECP2* related pathways^4^ influence the severity of a rare monogenic (X-linked) disease and can possibly even save individuals with a documented pathogenic variation from disease development^51^. In principle, patients could also have an unreported second mutation that could cause the effect either alone or through epistatic interaction. Another reason for misinterpretation of a variant may be due to linkage disequilibrium, where the causal mutation is not the reported one but another unreported.

For several variations, a high pathogenicity score was predicted but they were still documented in healthy individuals. This has been observed before in a girl with RTT who inherited a germline disease causing *MECP2* c.1160 C > T (P387L, NC_000023.11:g.154030668 G > A) variation from a healthy (!) father^52^. We found exactly this variant only in our RTT causing dataset (documented in ClinVar and RettBase), the annotation with the benign outcome was not added to one of these databases yet. These effects may contribute to the limited penetrance of some mutations. To unravel the different influences of *MECP2* variations in the context of an individual patient, we need to evaluate how genetic background (ancestry) can affect other process related genes. For this, genotype-phenotype databases with detailed phenotype capture will be highly important and data integration tools and methods must be developed to investigate this further.

There is a possibility that a gene carries more than one variation. Indeed, in our integrated dataset we found a total 54 individuals with multiple variants. However, multiple variants are difficult to predict, there may be positive or negative epistatic effects if these variants occur on the same allele or two mutations affecting the same codon, one cancelling out to another. All of these possibilities may lead to wrong classification of variants.

## Supplementary information

Supplementary Table 1

Supplementary Table 2

## Data Availability

Any custom code used to generate and analyse this dataset is openly available on Git-based repositories. For data FAIRification see https://github.com/stkenny/grefine-rdf-extension and https://github.com/LUMC-BioSemantics/rett-variant, for VEP data analysis see https://github.com/counsyl/hgvs, and https://gitlab.bsc.es/inb/fair-rett for _summary_plots and HGVS pipelines.
