# Supplementary material for: A catalogue of 863 Rett-syndrome-causing MECP2 mutations and lessons learned from data integration
Source: Sci Data. 2021 Jan 15;8:10. doi: 10.1038/s41597-020-00794-7 (PMC7810705; doi:10.1038/s41597-020-00794-7)
Supplement: Supplementary file 1 — Supplementary Table 1 [file 41597_2020_794_MOESM1_ESM.docx]

**Supplementary Table 1:** Annotation selection criteria for the RTT causing dataset

| **Database** | **RTT causing annotated with** |
| --- | --- |
| DECIPHER | Annotation with phenotypes commonly associated with RTT (intellectual disability, microcephaly, hypotonia, absence of speech...) |
| Maastricht Rett dataset | Annotation with Rett syndrome, Rett syndrome preserved speech variant, or congenital variant |
| ClinVar | Annotation with "Rett syndrome" or "X-linked mental retardation 13" (="male" Rett syndrome) or "encephalopathy" AND clinical significance is NOT benign |
| RettBase | Annotation with "Rett syndrome" (including variants and atypical forms) or "X-linked mental retardation, with MECP2 variation likely causing disorder" |
| KMD | Annotation with "Rett syndrome" or "Rett variant" |
