# Supplementary material for: A catalogue of 863 Rett-syndrome-causing MECP2 mutations and lessons learned from data integration
Source: Sci Data. 2021 Jan 15;8:10. doi: 10.1038/s41597-020-00794-7 (PMC7810705; doi:10.1038/s41597-020-00794-7)
Supplement: Supplementary file 2 — Supplementary Table 2 [file 41597_2020_794_MOESM2_ESM.docx]

**Supplementary Table 2:** MECP2 variations, which occur in both, RTT causing and benign dataset.

| **Chromosome variant NC_000023.11** | **cDNA variant NM_004992.3** | **Protein change** | **Variant type** | **Predicted effect** | **RTT causing reference found in** | **Benign reference found in** |
| --- | --- | --- | --- | --- | --- | --- |
| g.154030546C>T | c.1282G>A | G/S | Missense variant | tolerated/ benign | ClinVar | RB |
| g.154030639C>T | c.1189G>A | E/K | Missense variant | tolerated/ benign | ClinVar | RB |
| g.154030674G>T | c.1154C>A | P/H | Missense variant | tolerated/ benign | ClinVar | ClinVar |
| g.154030695G>C | c.1133C>G | A/G | Missense variant | deleterious/ benign | ClinVar and RB | RB |
| g.154030886G>A | c.942C>T | I | Synonymous | - | ClinVar | ClinVar and RB |
| g.154030988G>A | c.840C>T | A | Synonymous | - | ClinVar | RB |
| g.154031220G>A | c.608C>T | T/M | Missense variant | deleterious/ benign | ClinVar | ClinVar and RB |
| g.154031226G>A | c.602C>T | A/V | Missense variant | tolerated/ benign | ClinVar and KMD | RB |
| g.154031267G>T | c.561C>A | G | Synonymous | - | ClinVar | ClinVar |
| g.154031378C>G | c.450G>C | L | Synonymous | - | ClinVar | ClinVar |
| g.154031408C>T | c.420G>A | A | Synonymous | - | ClinVar | ClinVar |
| g.154031456G>C | c.378-6C>G | - | Splice region variant, intron variant | - | ClinVar | ClinVar |
| g.154031467delA | c.378-17delT | - | Intron variant | - | ClinVar | RB |
| g.154031492T>C | c.378-42A>G | - | Intron variant | - | ClinVar | ClinVar |
| g.154030632G>A | c.1196C>T | P/L | Missense variant | deleterious/ benign | Decipher | ClinVar and RB |
| g.154031133C>G | c.695G>C | G/A | Missense variant | tolerated/ benign | KMD | ClinVar and RB |
| g.154030696C>A | c.1132G>T | A/S | Missense variant | tolerated/ benign | RB | ClinVar |
| g.154030655_  154030660del6 | c.1168_1173del6 | PP/- | Inframe deletion | - | RB | RB |
| g.154030513C>T | c.1315G>A | A/T | Missense variant | tolerated low confidence/ benign | RB | ClinVar |
